# Adipose triglyceride lipase is involved in the mobilization of triglyceride and retinoid stores of hepatic stellate cells

**DOI:** 10.1016/j.bbalip.2015.02.017

**Published:** 2015-07

**Authors:** Ulrike Taschler, Renate Schreiber, Chandramohan Chitraju, Gernot F. Grabner, Matthias Romauch, Heimo Wolinski, Guenter Haemmerle, Rolf Breinbauer, Rudolf Zechner, Achim Lass, Robert Zimmermann

**Affiliations:** a Institute of Molecular Biosciences, University of Graz, Graz 8010, Austria; b Institute of Organic Chemistry, Graz University of Technology, Graz 8010, Austria

**Keywords:** Adipose triglyceride lipase, Hepatic stellate cells, Retinoids, Retinyl ester

## Abstract

Hepatic stellate cells (HSCs) store triglycerides (TGs) and retinyl ester (RE) in cytosolic lipid droplets. RE stores are degraded following retinoid starvation or in response to pathogenic stimuli resulting in HSC activation. At present, the major enzymes catalyzing lipid degradation in HSCs are unknown. In this study, we investigated whether adipose triglyceride lipase (ATGL) is involved in RE catabolism of HSCs. Additionally, we compared the effects of ATGL deficiency and hormone-sensitive lipase (HSL) deficiency, a known RE hydrolase (REH), on RE stores in liver and adipose tissue. We show that ATGL degrades RE even in the presence of TGs, implicating that these substrates compete for ATGL binding. REH activity was stimulated and inhibited by comparative gene identification-58 and G0/G1 switch gene-2, respectively, the physiological regulators of ATGL activity. In cultured primary murine HSCs, pharmacological inhibition of ATGL, but not HSL, increased RE accumulation. In mice globally lacking ATGL or HSL, RE contents in white adipose tissue were decreased or increased, respectively, while plasma retinol and liver RE levels remained unchanged. In conclusion, our study shows that ATGL acts as REH in HSCs promoting the degradation of RE stores in addition to its established function as TG lipase. HSL is the predominant REH in adipocytes but does not affect lipid mobilization in HSCs.

## Highlights

• ATGL possesses retinyl ester and triacylglycerol hydrolase activity.
• The lack of ATGL activity causes increased triacylglycerol and retinyl ester storage in hepatic stellate cells.
• ATGL acts as retinyl ester and triacylglycerol lipase in hepatic stellate cells.

## Graphical abstract

**Figure:**
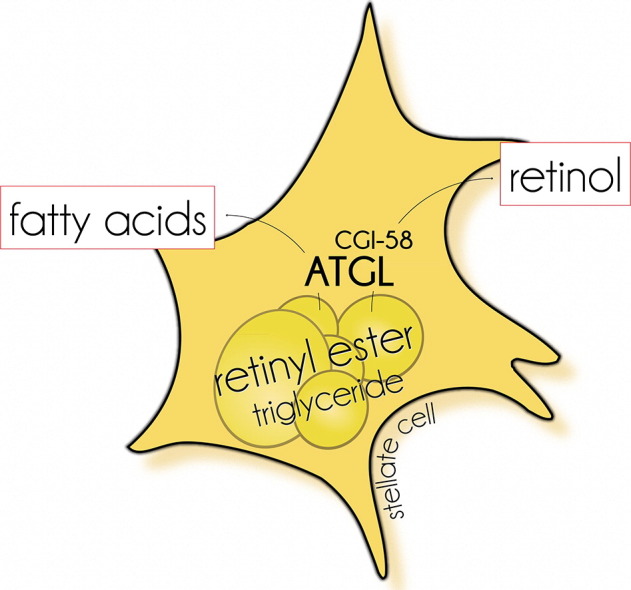

## 1 Introduction

Retinoids (vitamin A) are fat-soluble micronutrients with numerous important functions, including a role in vision, reproduction, immunity, and the development and maintenance of differentiated tissues [1]. Dietary retinol (ROH) is esterified with long-chain fatty acids (FAs), preferentially palmitic acid (PA), and stored in form of retinyl ester (RE). Although most tissues of the body contain trace amounts of RE, they are predominantly (up to 80%) stored in the liver where they are deposited in lipid droplets (LDs) of hepatic stellate cells (HSCs) [2]. Healthy individuals contain vitamin A reserves sufficient for an adequate supply of the body for up to several months. In times of insufficient vitamin A uptake, RE stores are mobilized to ensure a constant concentration of ~ 1 μM ROH in the circulation which is essential for normal body function [3]. In the circulation, ROH is attached to retinol-binding protein 4 (RBP4) and is transported to target tissues where it can be re-esterified or converted into its bioactive metabolites, which possess essential roles in vision and gene regulation. 11-*cis* retinaldehyde functions as the active chromophore in rhodopsin. All-*trans* and 9-*cis* retinoic acid interact with a number of nuclear receptors of the retinoic acid receptor (RAR) and the retinoid X receptor (RXR) family. These receptors function as ligand-activated transcription factors and regulate the expression of numerous genes [4,5].

The availability of ROH from endogenous stores is determined by the synthesis and hydrolysis of RE. In HSCs, ROH is esterified by the action of lecithin:retinol acyltransferase (LRAT). LRAT-deficient mice possess only trace amounts of RE in HSCs and are more susceptible to develop retinoid deficiency [6], demonstrating that this enzyme is required for efficient RE storage. Yet these mice exhibit elevated RE levels in white adipose tissue (WAT) and are capable of esterifying ROH in other tissues in an acyl-CoA-dependent reaction. Presumably, this reaction [acyl-CoA:retinol acyltransferase activity] is mediated by acyl-CoA:diacylglycerol acyltransferase 1, an enzyme with broad substrate specificity catalyzing the synthesis of triglycerides (TGs), diglycerides (DGs), and waxes [7]. The mobilization of ROH from RE stores requires the activity of enzymes possessing RE hydrolase (REH) activity. To date, very little is known about lipases involved in RE mobilization in HSCs and the molecular mechanisms regulating lipolysis in this cell type [7,8]. Yet it is reasonable to assume that RE mobilization is a tightly regulated process, since the liver is capable of keeping constant plasma ROH levels despite strong variations in dietary retinoid uptake.

Although a number of enzymes have been reported to hydrolyze RE *in vitro* [7], hormone-sensitive lipase (HSL) and retinal pigment epithelium 65 (RPE65) are the only known enzymes, which have been shown to affect retinoid metabolism *in vivo* [9,10]. However, HSL is hardly detectable in HSCs [11], and RPE65 is specifically expressed in retinal pigment epithelium, a specialized cell type controlling retinoid metabolism in the eye [12]. In the search for enzymes involved in HSCs lipid catabolism, we found that adipose triglyceride lipase [ATGL [13], identical to desnutrin and patatin-like phospholipase domain containing 2 (PNPLA2)] can hydrolyze RE in the presence of its co-activator comparative gene identification-58 [CGI-58, identical to α/β-hydrolase domain containing-5 [14]]. Our findings indicate that ATGL functions as REH in HSCs in addition to its established role in TG catabolism.

## 2 Materials and methods

### 2.1 Materials

All-*trans*-retinol (ROH), retinyl acetate (RAc), retinyl palmitate (RP), and fatty acid (FA)-free BSA were from Sigma-Aldrich (Taufkirchen, Germany).

### 2.2 Animals

Mice were maintained on a regular light–dark cycle (12 h light, 12 h dark) and kept *ad libitum* on a standard laboratory chow diet (Ssniff Spezialdiaeten, Soest, Germany, Vitamin A ~ 15,000 IU/kg) or on a Vitamin A-deficient diet (Ssniff, Vitamin A < 120 IU/kg). ATGL-ko and HSL-ko mice were generated by targeted homologous recombination as described previously [15,16]. Animals were 8–12 weeks of age. Female mice were used for all studies. Non-fasted animals were anesthetized with IsoFlo/Isoflurane (Abbott, Animal Health, Queenborough, Kent, UK) and euthanized by cervical dislocation. The study was approved by the ethics committee of the University of Graz and is in accordance with the Council of Europe Convention (ETS 123).

### 2.3 cDNA cloning of recombinant tagged proteins

The open reading frames (ORF) of murine PNPLA1, ATGL, and adiponutrin were cloned into pcDNA4™/HisMaxC (Invitrogen; Life Technologies, Carlsbad, USA), as described previously [17,18], and transfected into COS-7 cells (ATCC CRL-1651) using Metafectene (Biontex GmbH, Munich, Germany). PNPLA1 was cloned using the following primers: fw-5′-AAGAATTCGAACAGGTGTTCAAAGGAG-3′ and rev-5′-AACTCGAGTTAGGAGTTCTGGCCACTCACT-3′: for the expression of murine ATGL and CGI-58 in *Escherichia coli*, sequences encoding the ORF of ATGL and CGI-58 were inserted into the target vector pASK-IBA5plus (IBA, Goettingen, Germany) and transformed into *E. coli* as described [17].

### 2.4 Immunoblotting

Proteins of cell lysates or tissue homogenates were separated by SDS–PAGE according to their molecular weight using Tris/glycine as electrophoresis buffer and were transferred onto polyvinylidene fluoride membranes (Carl Roth GmbH, Karlsruhe, Germany) using CAPS buffer. After blocking, membranes were hybridized with respective primary antibodies. Membranes were washed, incubated with respective secondary horseradish-peroxidase (HRP)-conjugated antibody and detected using ECL2 Western blotting substrate (Thermo Scientific, Waltham, MA). Antibodies used were rabbit anti-HSL, rabbit anti-phospho-HSL (S660), and rabbit anti-GAPDH (all from Cell Signaling, Danvers, MA, USA), rabbit anti-CGI-58 (Abnova, Heidelberg), mouse anti-β-Actin (Santa Cruz, Santa Cruz, CA, USA), rabbit anti-alpha smooth muscle actin (α-SMA, Pierce, Thermo Scientific), HRP-linked sheep-anti mouse antibody (GE Healthcare Amersham, Buckinghamshire, UK), and HRP-linked rat-anti rabbit antibody (Dako, Glostrup, Denmark).

### 2.5 Isolation of HSCs

**Fig. S1:**
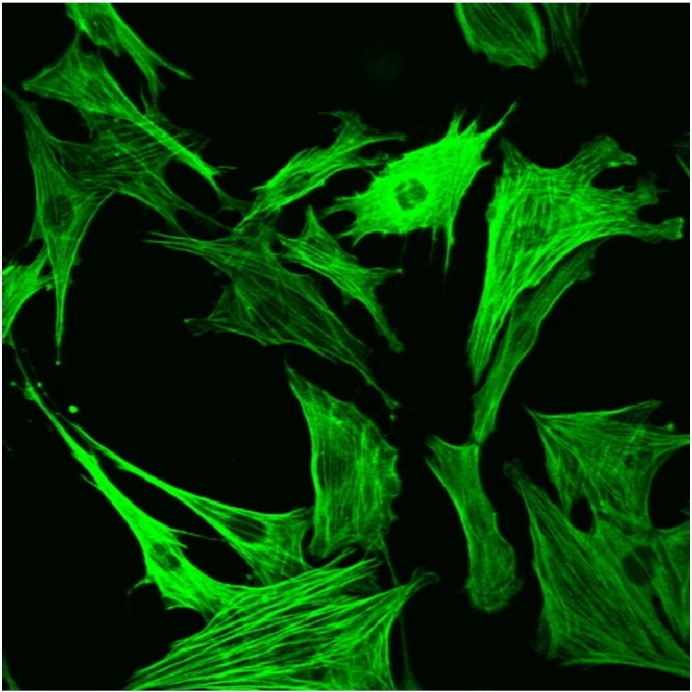
Immunohistochemistry of HSCs. Hepatic non-parenchymal cells were cultured for 10 days on plastic dishes resulting in the activation and proliferation of HSCs. Cultured cells stained positive for α-SMA indicating the presence of activated HSCs.

HSCs were isolated according to the method of Blomhoff et al. [19] with some modifications. Briefly, livers of anaesthetized mice were perfused via the portal vein using Krebs–Henseleit buffer (KHB, without Ca^2 +^ and SO_4_^2 −^) followed by perfusion with KHB containing 0.15 mg/ml collagenase type II (Worthington Biochemical Corporation, Lakewood, NJ), 0.1 mg/ml Pronase E (Merck, Darmstadt, Germany), 2% BSA, and 0.1 mM CaCl_2_. Thereafter, livers were excised, disrupted, passed through a metal sieve, and filtered through a 70 μm nylon cell strainer (BD Biosciences, San Jose, CA). Parenchymal cells were separated from non-parenchymal cells (NPCs) by centrifugation (50 ×*g*, 3 min, 4 °C). The supernatant was used for the isolation of HSCs using OptiPrep™ self-forming density gradient solutions (Axis-Shield PoC AS, Rodeløkka, Norway) as described previously [20]. After centrifugation, HSCs were collected, washed with PBS, and used for western blotting analyses. For cell culture experiments, total NPCs were seeded into cell culture dishes in Dulbecco's modified Eagle medium (DMEM) containing 20% fetal calf serum (FCS), 100 μg/ml primocin, and antibiotics. After 2 days in culture, cells were trypsinized and seeded again [selective detachment according to Trøen et al. [21]]. After 10 days in culture, ~ 90% of cells stained positive for α-smooth muscle actin (α-SMA), indicating the presence of activated stellate cells. A representative stain for α-SMA is now shown in Fig. S1.

### 2.6 Lipid accumulation in primary HSCs

HSCs were seeded into 6-well plates and cultured for 10 days in DMEM media containing 20% FCS, 100 μg/ml primocin, and antibiotics. ROH (5 μM) was added to the medium to prevent complete loss of RE stores [21]. Cells were loaded for 24 h with 20 μM ROH and 50 μM palmitic acid (PA) complexed to BSA. Thereafter, cells were starved in DMEM media containing 2% FA-free BSA. At indicated time points, lipids were extracted with hexane:isopropanol (3:2; 0.5 mM butylated hydroxytoluene (BHT)).

### 2.7 Analysis of tissue and plasma ROH and RE by HPLC

Retinoids of liver, WAT, and plasma samples were extracted with ice-cold hexane:methanol:PBS (5:1:1; 0.5 mM BHT and 100pmol RAc/sample as internal standard). Hexane phase was dried, and lipids were dissolved in methanol and subjected to HPLC analysis. Retinoids were separated on a reverse phase pro-C18 column (250 × 4.6 mm; 12 nm, S-5 μm; YMC Europe, Dinslaken, Germany) using a gradient of methanol:toluene at a flow rate of 1.0 ml/min. The HPLC system consisted of Waters e2695 Separation Module, UV/VIS 2489 Detector, and a Multi-λ Fluorescence 2475 Detector (Waters, Millford, MA). For the detection of ROH and RE, excitation was set at 325 nm and emission at 490 nm. ROH eluted at a retention time of 5.3 min, whereas RE eluted as a single peak at a retention time of 12.2 min. ROH and RE were quantified by comparison with the internal standard.

### 2.8 Determination of enzyme activities

The determination of TG hydrolase activities was performed using [9,10-^3^H]-triolein (TO) (PerkinElmer Life Sciences) as radioactive tracer as described [22]. The substrate for the determination of REH activities consisted of RP and phosphatidylcholine (molar ratio 1:0.9) in 0.1 M potassium phosphate buffer (pH 7.0). Substrate was prepared by sonication (Virsonic 475, Virtis, Gardiner, NJ) on ice and adjusted to 5% FA-free BSA. To analyze REH activities, 100 μl cell or tissue lysates (100 μg total protein) were incubated with 100 μl lipid substrate at 37 °C for 1 h in a shaking water bath. In some cases purified G0S2 [23] was added to the incubation mixture. The reaction was terminated by the addition of ice-cold methanol (containing 0.5 mM BHT and 100pmol RAc/sample as internal standard) and hexane. Retinoids were extracted, and the hexane phase was dried. The release of ROH was determined by HPLC.

### 2.9 Determination of TG and RE content of HSCs

Cells were washed with PBS, then cellular lipids were extracted with hexane:isopropanol (3:2), and the hexane phase was dried and dissolved in methanol for the determination of RE content (see above). For TG measurement, lipids were dissolved in chloroform (containing 1% Triton X-100), and the solvent was evaporated again. Lipids were solubilized in deionized water by vigorous shaking, and TG content was determined using a commercial Kit (Infinity^TM^ triglycerides, Thermo Scientific).

## 3 Immunohistochemistry of HSC

Cells were seeded at a density of 1 × 10^5^/cm^2^ onto Lab-Tek chamber slights (Thermo Scientific) and cultured overnight in DMEM (4.5 mg/ml glucose, 20% FCS and 100 μg/ml primocin). After 24 h, cells were washed with PBS and fixed with ice-cold methanol-acetone (1:1) for 10 min at − 20 °C. The solution was carefully removed, and cells were washed again with PBS. HSCs were identified by staining with monoclonal anti α-SMA. Briefly, cells were incubated with monoclonal α-SMA antibody for 1 h at room temperature (RT). Slides were washed with PBS followed by 30 min incubation with the secondary antibody anti-mouse FITC (fluorescein isothiocyanate). After washing with ddH_2_O and ethanol, slides were dried in the dark and mounted with Moviol (Hoechst, Frankfurt, Germany). Slides were immediately subjected to microscopy (Zeiss LSM 510, Oberkochen, Germany) and FITC-fluorescence was detected at 488 nm.

### 3.1 Microscopy

Coherent anti-Stokes Raman scattering (CARS) microscopy of primary HSCs was performed using a commercial setup consisting of a picosecond laser source and an optical parametric oscillator (*pico*Emerald; APE, Germany; HighQ Laser, Austria) integrated into a Leica SP5 confocal microscope (Leica Microsystems, Inc.). The detection of the CARS signal was achieved using 650/210 and 770/SP emission filters. The microscope was equipped with a non-descanned detector for acquisition of signals in forward (F-) CARS mode. To detect neutral lipids, the laser was tuned to 2845 cm^− 1^, thus enabling imaging of CH_2_ symmetric stretching vibrations. For imaging a Leica 1.25 NA, 40 × oil objective was used.

### 3.2 Protein determination

Protein concentrations were determined by Bio-Rad protein (Bio-Rad) or BCA protein assay (Thermo Scientific) kits according to manufacturer's instructions using BSA as standard.

### 3.3 Statistical analyses

Values are means ± standard deviation (SD). Statistical significance was determined by Student's unpaired *t*-test (two-tailed). Group differences were considered statistically significant for *p < 0.05, **p < 0.01, and ***p < 0.001.

## 4 Results

### 4.1 ATGL hydrolyzes RE *in vitro*

**Fig. 1:**
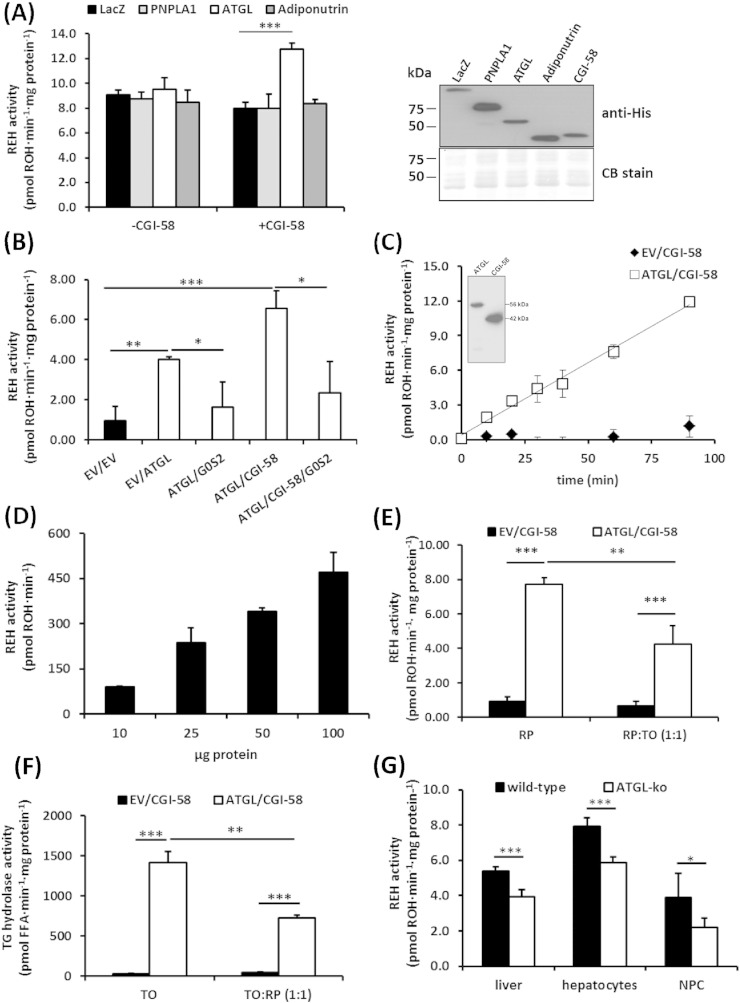
ATGL exhibits REH activity. (A) REH activities were determined in COS-7 cell lysates containing PNPLA1, ATGL, or adiponutrin as well as upon the addition of lysates containing CGI-58. Lysates containing LacZ were used as negative control. Insert shows the expression of respective enzymes by Western blotting analysis. Coomassie blue (CB) stain was used as loading control. (B) REH activities of *E. coli* lysates containing ATGL, CGI-58, or of empty vector transformed cells (EV) were determined in the presence or absence of purified G0S2. REH activities (release of ROH) of bacterial lysates containing ATGL and CGI-58 were determined at different time points (C), and with increasing protein concentrations (D) using RP emulsified with phospholipids (PL) as substrate. Insert in C shows the expression of ATGL and CGI-58 in *E. coli* by Western blotting analysis. (E) REH activities of *E. coli* lysates containing ATGL and CGI-58 or of EV transformed cells were determined using RP or an equimolar mixture of RP and triolein (TO) emulsified with PL as substrates. (F) As in E but using TO or an equimolar mixture of TO and RP emulsified with PL as substrates. (G) REH activities were determined in liver lysates and cell lysates of primary hepatocytes and non-parenchymal cells (NPCs) of wild-type and ATGL-ko mice using RP emulsified with PL as substrate. ROH release was determined by HPLC (*n* = 5–7/genotype). All data are representative for at least two independent experiments performed in triplicates and are presented as means ± SD (*p < 0.05; **p < 0.01; ***p < 0.001).

Recent evidence suggests that human adiponutrin (PNPLA3) is able to hydrolyze RE *in vitro* [24]. Thus, we investigated whether mouse PNPLA3 and the closest mouse homologues, PNPLA1 and ATGL (PNPLA2), are capable of hydrolyzing RE. REH activity assays were performed using lysates of COS-7 cells expressing murine PNPLA1, ATGL, and adiponutrin. We did not detect REH activity for any of these enzymes. However, the addition of CGI-58, the co-activator protein of ATGL, to the cell lysates increased REH activity 1.5-fold in ATGL containing lysates. No activation was observed for PNPLA1 and adiponutrin (Fig. 1A). To confirm REH activity of ATGL, we expressed ATGL in *E. coli* and determined REH activities in these lysates in the absence or presence of CGI-58 and the ATGL-specific inhibitory protein G0/G1 switch gene 2 (G0S2) [25]. The expression of ATGL-elevated REH activity in bacterial lysates and the addition of CGI-58 further increased this activity by 2.7-fold. Conversely, G0S2 completely abolished ATGL's REH activity (Fig. 1B). Further experiments revealed that ATGL degrades RE in a time- and dose-dependent manner (Fig. 1C, D).

RE and TGs are present at similar concentrations in LDs of HSCs [2,26–28]. To investigate whether ATGL is capable of degrading RE in the presence of TG and *vice versa*, we prepared mixed substrates containing equimolar concentrations of TO and RP and incubated them with lysates containing ATGL and CGI-58. The addition of TO to the RP substrate reduced ATGL's REH activity by ~ 50% (Fig. 1E). Notably, TG hydrolase activity of ATGL was also reduced by ~ 60% in the presence of RP suggesting that these substrates compete for ATGL binding (Fig. 1F).

To investigate whether ATGL-ko mice exhibit reduced neutral REH activity in liver, we measured REH activity in lipid-poor lysates (20.000 ×*g* infranatant) prepared from total liver, from isolated hepatocytes, or from isolated non-parenchymal cells (NPCs). In comparison to the wild-type controls, REH activities in total liver, hepatocytes, and NPCs of ATGL-ko mice were decreased by 27%, 26%, and 44%, respectively (Fig. 1G). These observations indicate that ATGL contributes to the neutral REH activity detected in liver preparations, but is clearly not the only enzyme capable of catalyzing this reaction.

### 4.2 ATGL is expressed in HSCs and affects cellular RE and TG contents

**Fig. 2:**
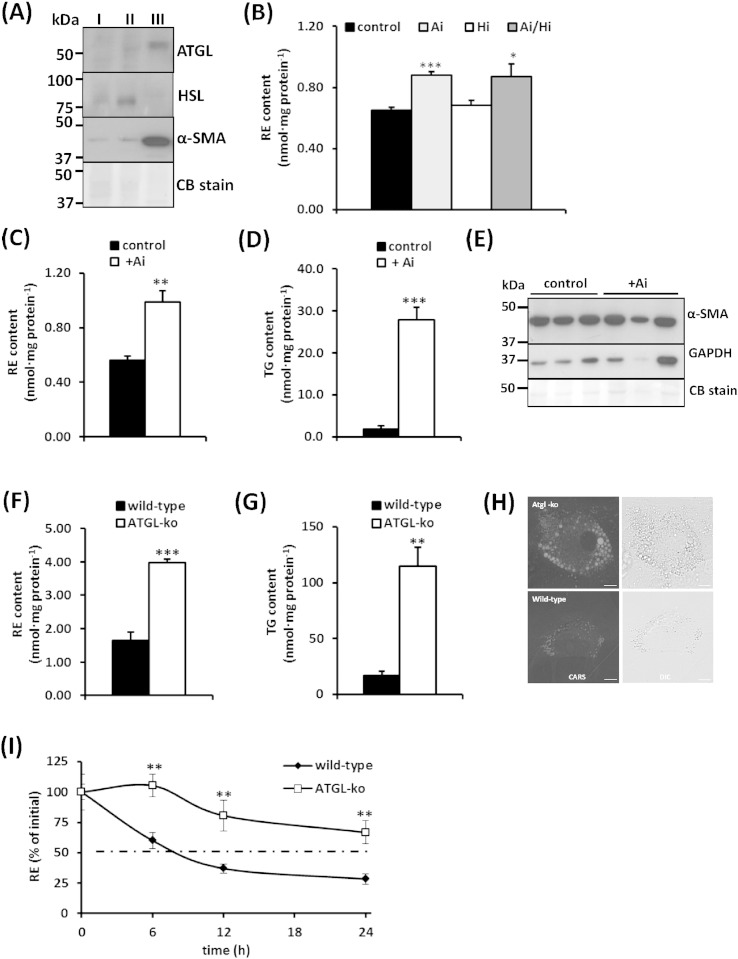
ATGL is expressed in HSCs and affects cellular RE and TG stores. (A) Western blotting analysis of cell lysates of mouse primary hepatocytes (I), total NPCs (II), and isolated HSCs (III) showing the expression of ATGL, HSL, and α-SMA. Coomassie blue (CB) stain was used as loading control. (B) Primary HSCs were isolated and cultured for 10 days in DMEM containing 5 μM ROH. Then cells were maintained in the presence of DMSO (control), 40 μM Atglistatin (Ai), 20 μM Hi-76-0079 (Hi), or the combination thereof. After 12 h, lipids were extracted, and the cellular RE content was determined by HPLC. (C, D) Primary HSCs were isolated and cultured for 10 days in the absence or presence of Ai (daily, 40 μM). Lipids were extracted and the cellular RE content was determined by HPLC (C). Cellular TG content was determined using a commercial kit (D). (E) Expression of α-SMA was determined by Western blotting analysis, GAPDH and CB stain were used as loading control. (F, G) RE and TG contents of HSCs from wild-type and ATGL-ko mice cultured in DMEM containing 20% FCS and 5 μM ROH for 10 days. RE and TG contents were determined as in (C, D). (H) CARS microscopy analyses of cultured HSCs derived from wild-type and ATGL-ko mice. (I) Primary HSCs from wild-type and ATGL-ko mice were loaded for 24 h with 20 μM ROH and 50 μM PA. Then cells were starved in serum-free DMEM containing 2% BSA. At indicated time points, lipids were extracted, and the cellular RE content was determined by HPLC. Data are representative for at least two independent experiments and are presented as means ± SD (*p < 0.05;**p < 0.01; ***p < 0.001).

ATGL protein expression was detected in HSCs (Fig. 2A, lane III) but remained below detection limit in the hepatocyte or total NPC fraction (Fig. 2A, lane I and II, respectively). HSL is a major REH in WAT but only poorly expressed in the liver [11]. We could not detect HSL protein expression in HSCs (Fig. 2A, lane III), whereas HSL was detected in the hepatocyte and total NPC fraction (Fig. 2A, lane I and II, respectively). To investigate whether inhibition of ATGL and/or HSL affects RE catabolism in cultured wild-type HSCs, we inhibited respective lipases with ATGL and/or HSL specific inhibitors, Atglistatin (Ai) [29] and/or Hi-76-0079 (Hi) [30], respectively. After incubation of primary HSCs for 12 h in the presence of 5 μM ROH, Ai caused a 30% increase of cellular RE content, while inhibition of HSL did not change RE levels (Fig. 2B). These observations indicate that ATGL, but not HSL, contributes to RE mobilization in HSCs. In accordance with our observation, previous studies reported high ATGL mRNA levels in HSCs [11,31]. When we cultured primary HSCs for 7 days in the presence of Ai, we determined a 1.7-fold increase in RE content and a 14-fold increase in TG content (Fig. 2C, D). This treatment did not affect the expression of α-SMA (Fig. 2E), suggesting that the lack of ATGL activity does not influence HSC activation. CARS microscopy analysis of HSCs isolated from wild-type and ATGL-ko mice, allowing the label-free detection of lipid stores, revealed larger LDs in HSCs lacking ATGL (Fig. 2H). RE and TG contents of ATGL-ko HSCs were increased 2.4-fold (Fig. 2F) and 6.7-fold (Fig. 2G), respectively, in comparison to wild-type HSCs. Furthermore, a time course experiment revealed that isolated wild-type HSCs lost 50% of their RE stores within ~ 6 h, while ATGL-ko cells retained almost 70% of their initial stores after 24 h (Fig. 2I). Thus, ATGL deficiency substantially attenuates RE mobilization in HSCs.

### 4.3 ATGL- and HSL-deficient mice exhibit unchanged liver and plasma ROH and RE levels but decreased and increased RE stores in adipose tissue, respectively

**Fig. S2:**
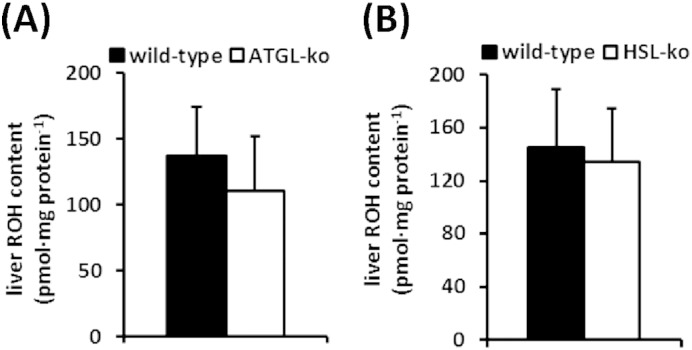
Liver ROH concentrations of wild-type and mutant mice. Wild-type, ATGL-ko, and HSL-ko mice were fed a retinoid-free diet for 3 weeks. Then animals were sacrificed and liver ROH content was determined by HPLC (*n* = 5–7/genotype).

**Fig. 3:**
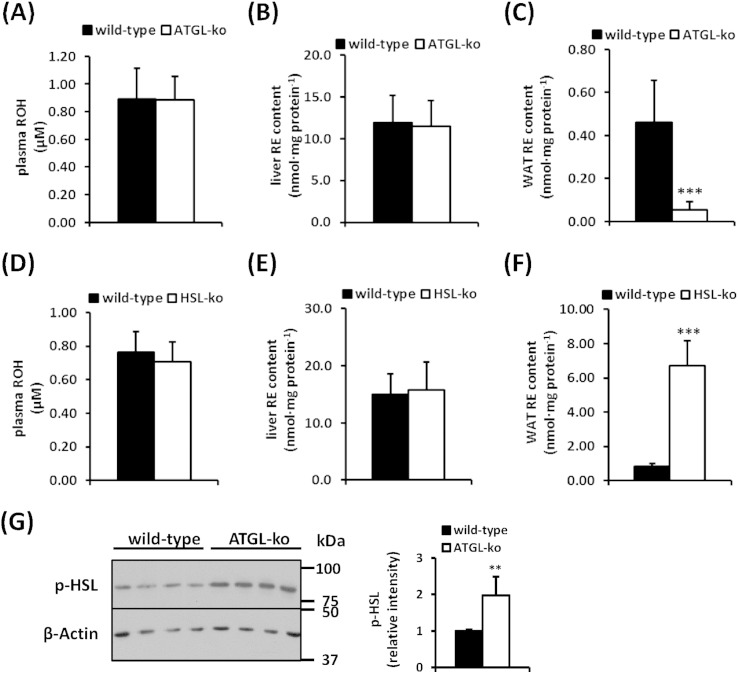
Plasma, liver, and WAT ROH and RE concentrations of wild-type and mutant mice. Wild-type, ATGL-ko, and HSL-ko mice were fed a retinoid-free diet for 3 weeks. Then animals were sacrificed and plasma ROH (A, D), liver RE (B, E), and WAT RE (C, F) contents were determined by HPLC (*n* = 5–7/genotype). (G) HSL phosphorylation was determined in 1,000 ×*g* supernatant of wild-type and ATGL-ko WAT. Expression of β-actin was used as loading control. Insert depicts densitometric quantitation of HSL phosphorylation as normalized to β-actin. Data are presented as means ± SD (**p < 0.01; ***p < 0.001).

In order to investigate whether the lack of ATGL or HSL affect RE metabolism *in vivo*, we compared plasma, hepatic, and adipose tissue ROH and RE levels of wild-type and ATGL-ko mice. To exclude the contribution of dietary vitamin A, mice were fed a retinoid-deficient diet for 3 weeks. As shown in Fig. 3A, circulating ROH concentrations did not differ between groups and we did not observe differences in hepatic RE content between wild-type and ATGL-ko mice (Fig. 3B). Interestingly, however, WAT RE stores of ATGL-ko mice were depleted as compared to wild-type mice (Fig. 3C). We also could not observe any differences in circulating ROH levels or hepatic RE stores in HSL-ko mice (Fig. 3D, E). In accordance with published data [9], these mice showed an 8-fold increase in WAT RE content (Fig. 3F), implicating a central role of HSL in WAT RE catabolism. Furthermore, hepatic ROH levels were not different between wild-type and ATGL-ko or HSL-ko mice (Fig. S2). Notably, we found increased HSL phosphorylation in ATGL-ko WAT (Fig. 3G), indicative for increased HSL activity which may explain the depletion of WAT RE stores in ATGL-ko mice.

## 5 Discussion

ATGL deficiency leads to impaired lipolysis in many tissues, and is associated with systemic TG accumulation in rodents and humans [32]. Several studies demonstrated that ATGL is also a key TG lipase in hepatocytes [33,34]. Moreover, the enzyme is expressed in hepatic and non-hepatic resident cell types such as quiescent and activated HSCs, Kupffer cells, and macrophages [11,35]. Here we demonstrate that cultured mouse HSCs lacking ATGL exhibit a substantial increase in TG storage confirming an important role of the enzyme in this cell type. We originally reported that ATGL catalyzes specifically the hydrolysis of TG into DG and FA and has no detectable REH activity [13]. However, these observations were made prior to the discovery of CGI-58, the activator protein of ATGL [14]. Present data demonstrate that ATGL possesses REH activity that is stimulated by CGI-58 and inhibited by G0S2, the physiological activator and inhibitor proteins of ATGL. Although ATGL degrades TGs at a much higher rate than RE, REH activity was clearly detected in the presence of equimolar concentrations of TG. Notably, the addition of RE to the substrate inhibited TG hydrolase activity of ATGL, suggesting that RE and TGs compete for ATGL binding. Moriwaki et al. [28] reported the lipid composition of lipid droplets isolated from primary rat HSCs as follows: 39.5% RE, 31.7% TG, 15.4% cholesteryl ester, 6.3% phospholipid, 4.7% cholesterol, and 2.4% FFAs. Based on our *in vitro* substrate competition assays, this lipid droplet composition enables ATGL to hydrolyze both RE and TGs.

The lack of ATGL in cultured primary HSCs attenuates RE and TG degradation *in vitro*. This observation provides new insights into the molecular mechanisms of lipolysis in HSCs. It will be highly interesting to examine whether RE degradation in these cells is activated or inactivated by hormonal signals known to control lipolysis in adipocytes [36]. However, ATGL is clearly not the only enzyme involved in RE hydrolysis. ATGL deficiency in mice neither caused RE accumulation in the liver nor affected circulating ROH levels, suggesting a redundant enzyme system catalyzing RE degradation in HSCs. Published data indicate that RPE65, HSL, and human PNPLA3, as well as a number of other enzymes, including bile-salt activated carboxylester lipase, rat carboxylesterases 2, 3, 4, and 10 [8], are capable of hydrolyzing RE *in vitro*. Recently, we reported that mouse esterase 22 (Es22, the orthologue of rat carboxylesterase 3) is a potent REH, which is highly expressed in hepatocytes and not present in HSCs. Es22 is located at the ER, implicating that it rather counteracts RE formation at the ER than mobilizing cytosolic RE depots [20]. Interestingly, a patatin-domain containing protein named GS2 (gene sequence 2, annotated as PNPLA4), which exhibits homology to ATGL, was also shown to possess REH activity [37]. GS2 was originally identified as transacylase and lipase in keratinocytes. The human enzyme was reported to hydrolyze RE and TGs as well as to catalyze acyl-CoA-dependent and acyl-CoA-independent ROH esterification. GS2 is not encoded by the mouse genome, and the rat orthologue of the human enzyme does not share these activities [38]. Thus, it is reasonable to assume that, in addition to ATGL, one or more of these RE-degrading enzymes are involved in retinoid metabolism in HSCs. Yet their role in retinoid metabolism *in vivo* remains to be investigated. Furthermore, RE storage in HSCs is strongly dependent on LRAT activity [39]. To date, it is unclear if decreased RE synthesis (e.g. decreased LRAT activity) in ATGL-deficient HSCs counteracts RE accumulation, and that is why these animals do not exhibit increased liver RE content.

WAT contains the second largest RE depot of the body [40]. Interestingly, we observed depletion of WAT RE stores in ATGL-ko animals. WAT expresses HSL, which acts as major REH in adipocytes [9] and is the rate-limiting enzyme in the degradation of DGs [15,41]. Several studies demonstrate that HSL deficiency is associated with loss of WAT and reduced activity of peroxisome proliferator-activated receptor (PPAR)-γ, a crucial adipogenic transcription factor [42–44]. Ström *et al.* [9] proposed that defective ROH mobilization in HSL-ko adipocytes might be responsible for the lean phenotype and for the down-regulation of PPARγ target genes in HSL-ko mice since changes in retinoid levels can affect adipocyte survival and the activation of PPARγ/RXR heterodimers. In accordance with this report, we detected RE accumulation in WAT of HSL-ko mice confirming its important role in RE degradation in adipocytes. However, HSL is not expressed in HSCs, and we could not observe an increase in HSC RE content in response to pharmacological HSL inhibition. These observations implicate that HSL does not play a role in lipid catabolism of HSCs.

It is important to note that ATGL deficiency in mice is also associated with a down-regulation of PPARα- and PPARδ-target genes in tissues with high energy consumption [45,46]. Since PPARs have a prominent role in the transcriptional regulation of genes involved in FA uptake and oxidation, ATGL deficiency is associated with impaired mitochondrial respiration. Mechanistically, it has been proposed that ATGL generates long-chain FAs which could directly serve as ligands for PPARs or are required for the synthesis of ligands. In addition, the activation of PPARs is dependent on the formation of a heterodimer with RXR, which requires retinoic acid as ligand. Thus, ATGL could influence PPAR activity by increasing the cellular concentration of both FAs and ROH. It is also interesting to note that G0S2, the physiological inhibitor of ATGL, was identified as retinoic acid target gene and therefore might be involved in the regulation of cellular retinoid levels via inhibition of ATGL activity.

The lipolytic process in HSCs clearly has pathophysiological relevance since HSCs transform into a myofibroblast-like cell type in response to pathogenic signals. These activated cells are characterized by the loss of lipid stores and high expression of α-SMA. Activated HSCs start proliferating and are centrally involved in the synthesis and remodeling of the extracellular matrix in fibrosis [47]. It is believed that a treatment leading to the reduction of the fibrogenic or proliferative characteristics of HSCs can counteract the progression of liver disease [48]. Several studies have addressed the question how vitamin A influences liver fibrosis and HSC activation [49]. These studies are difficult to interpret since retinoids prevented or promoted liver disease, depending on the model system and on the experimental conditions. On the one hand, retinoids repress activation of HSCs [50–52], and vitamin A deficiency potentiates carbon tetrachloride-induced liver fibrosis in rats [53], suggesting a protective role of hepatic retinoid stores. On the other hand, the absence of RE stores in LRAT-ko mice does not promote fibrosis but appears to protect the chronically injured liver from developing tumors [39]. Moreover, excess dietary retinoids can cause liver damage [54], and it has been suggested that the rapid loss of retinoid stores can lead to toxic concentrations of bioactive retinoids, thereby promoting cell death and disease development [49,55]. Our studies suggest that a 7-day treatment of HSCs with ATGL inhibitor does not affect the expression of myofibroblast marker α-SMA *in vitro*. In future studies, it will be important to investigate how ATGL deficiency affects the fibrogenic and proliferative characteristics of HSCs *in vivo*. Published data suggest a protective role of ATGL against steatosis and hepatic inflammation [56].

In conclusion, our study provides novel insights into the molecular mechanisms regulating retinoid homeostasis. In addition to its established function as TG lipase, ATGL acts as REH in HSCs promoting the degradation of RE stores. HSL is the predominant REH in adipocytes but does not affect lipid mobilization in HSCs.

## Abbreviations

α-SMA: α-smooth muscle actin
ATGL: adipose triglyceride lipase
BHT: butylated hydroxytoluene
BSA: bovine serum albumin
CARS: coherent anti-Stokes Raman scattering
CGI-58: comparative gene identification-58
DG: diglycerides
DMEM: Dulbecco's modified Eagle medium
Es22: esterase 22
FA: fatty acid
FCS: fetal calf serum
GAPDH: glycerinaldehyd-3-phosphat-dehydrogenase
G0S2: G0/G1 switch gene 2
HSC: hepatic stellate cell
HSL: hormone-sensitive lipase
LD: lipid droplet
LRAT: lecithin:retinol acyltransferase
NPC: non-parenchymal cell
PA: palmitic acid
PL: phospholipids
PNPLA: patatin-like phospholipase domain containing
PPAR: peroxisome proliferator-activated receptor
RAc: retinyl acetate
RAR: retinoic acid receptor
RE: retinyl ester
REH: retinyl ester hydrolase
ROH: retinol
RP: retinyl palmitate
RPE65: retinal pigment epithelium 65
RXR: retinoid X receptor
TG: triglyceride
TO: triolein
WAT: white adipose tissue

The following are the supplementary data related to this article.

## Conflict of interest

The authors declare no conflict of interest.

## References

[1] D'Ambrosio D.N., Clugston R.D., Blaner W.S. (2011). Vitamin A metabolism: an update. Nutrients.

[2] Blaner W.S., O'Byrne S.M., Wongsiriroj N., Kluwe J., D'Ambrosio D.M., Jiang H., Schwabe R.F., Hillman E.M., Piantedosi R., Libien J. (2009). Hepatic stellate cell lipid droplets: a specialized lipid droplet for retinoid storage. Biochim. Biophys. Acta.

[3] Blomhoff R., Blomhoff H.K. (2006). Overview of retinoid metabolism and function. J. Neurobiol..

[4] Ziouzenkova O., Plutzky J. (2008). Retinoid metabolism and nuclear receptor responses: New insights into coordinated regulation of the PPAR-RXR complex. FEBS Lett..

[5] Amann P.M., Eichmuller S.B., Schmidt J., Bazhin A.V. (2011). Regulation of gene expression by retinoids. Curr. Med. Chem..

[6] O'Byrne S.M., Wongsiriroj N., Libien J., Vogel S., Goldberg I.J., Baehr W., Palczewski K., Blaner W.S. (2005). Retinoid absorption and storage is impaired in mice lacking lecithin:retinol acyltransferase (LRAT). J. Biol. Chem..

[7] Schreiber R., Taschler U., Preiss-Landl K., Wongsiriroj N., Zimmermann R., Lass A. (2012). Retinyl ester hydrolases and their roles in vitamin A homeostasis. Biochim. Biophys. Acta.

[8] Harrison E.H. (2000). Lipases and carboxylesterases: possible roles in the hepatic utilization of vitamin A. J. Nutr..

[9] Strom K., Gundersen T.E., Hansson O., Lucas S., Fernandez C., Blomhoff R., Holm C. (2009). Hormone-sensitive lipase (HSL) is also a retinyl ester hydrolase: evidence from mice lacking HSL. FASEB J..

[10] Moiseyev G., Takahashi Y., Chen Y., Gentleman S., Redmond T.M., Crouch R.K., Ma J.X. (2006). RPE65 is an iron(II)-dependent isomerohydrolase in the retinoid visual cycle. J. Biol. Chem..

[11] Mello T., Nakatsuka A., Fears S., Davis W., Tsukamoto H., Bosron W.F., Sanghani S.P. (2008). Expression of carboxylesterase and lipase genes in rat liver cell-types. Biochem. Biophys. Res. Commun..

[12] Wolf G. (2005). Function of the protein RPE65 in the visual cycle. Nutr. Rev..

[13] Zimmermann R., Strauss J.G., Haemmerle G., Schoiswohl G., Birner-Gruenberger R., Riederer M., Lass A., Neuberger G., Eisenhaber F., Hermetter A., Zechner R. (2004). Fat mobilization in adipose tissue is promoted by adipose triglyceride lipase. Science.

[14] Lass A., Zimmermann R., Haemmerle G., Riederer M., Schoiswohl G., Schweiger M., Kienesberger P., Strauss J.G., Gorkiewicz G., Zechner R. (2006). Adipose triglyceride lipase-mediated lipolysis of cellular fat stores is activated by CGI-58 and defective in Chanarin-Dorfman Syndrome. Cell Metab..

[15] Haemmerle G., Zimmermann R., Hayn M., Theussl C., Waeg G., Wagner E., Sattler W., Magin T.M., Wagner E.F., Zechner R. (2002). Hormone-sensitive lipase deficiency in mice causes diglyceride accumulation in adipose tissue, muscle, and testis. J. Biol. Chem..

[16] Haemmerle G., Lass A., Zimmermann R., Gorkiewicz G., Meyer C., Rozman J., Heldmaier G., Maier R., Theussl C., Eder S., Kratky D., Wagner E.F., Klingenspor M., Hoefler G., Zechner R. (2006). Defective lipolysis and altered energy metabolism in mice lacking adipose triglyceride lipase. Science.

[17] Nagy H.M., Paar M., Heier C., Moustafa T., Hofer P., Haemmerle G., Lass A., Zechner R., Oberer M., Zimmermann R. (2014). Adipose triglyceride lipase activity is inhibited by long-chain acyl-coenzyme A. Biochim. Biophys. Acta.

[18] Kumari M., Schoiswohl G., Chitraju C., Paar M., Cornaciu I., Rangrez A.Y., Wongsiriroj N., Nagy H.M., Ivanova P.T., Scott S.A., Knittelfelder O., Rechberger G.N., Birner-Gruenberger R., Eder S., Brown H.A., Haemmerle G., Oberer M., Lass A., Kershaw E.E., Zimmermann R., Zechner R. (2012). Adiponutrin functions as a nutritionally regulated lysophosphatidic acid acyltransferase. Cell Metab..

[19] Blomhoff R., Berg T. (1990). Isolation and cultivation of rat liver stellate cells. Methods Enzymol..

[20] Schreiber R., Taschler U., Wolinski H., Seper A., Tamegger S.N., Graf M., Kohlwein S.D., Haemmerle G., Zimmermann R., Zechner R., Lass A. (2009). Esterase 22 and beta-glucuronidase hydrolyze retinoids in mouse liver. J. Lipid Res..

[21] Troen G., Nilsson A., Norum K.R., Blomhoff R. (1994). Characterization of liver stellate cell retinyl ester storage. Biochem. J..

[22] Schweiger M., Eichmann T.O., Taschler U., Zimmermann R., Zechner R., Lass A. (2014). Measurement of Lipolysis. Methods Enzymol..

[23] Schweiger M., Paar M., Eder C., Brandis J., Moser E., Gorkiewicz G., Grond S., Radner F.P., Cerk I., Cornaciu I., Oberer M., Kersten S., Zechner R., Zimmermann R., Lass A. (2012). G0/G1 switch gene-2 regulates human adipocyte lipolysis by affecting activity and localization of adipose triglyceride lipase. J. Lipid Res..

[24] Pirazzi C., Valenti L., Motta B.M., Pingitore P., Hedfalk K., Mancina R.M., Burza M.A., Indiveri C., Ferro Y., Montalcini T., Maglio C., Dongiovanni P., Fargion S., Rametta R., Pujia A., Andersson L., Ghosal S., Levin M., Wiklund O., Iacovino M., Boren J., Romeo S. (2014). PNPLA3 has retinyl-palmitate lipase activity in human hepatic stellate cells. Hum. Mol. Genet..

[25] Yang X., Lu X., Lombes M., Rha G.B., Chi Y.I., Guerin T.M., Smart E.J., Liu J. (2010). The G(0)/G(1) switch gene 2 regulates adipose lipolysis through association with adipose triglyceride lipase. Cell Metab..

[26] Yamada M., Blaner W.S., Soprano D.R., Dixon J.L., Kjeldbye H.M., Goodman D.S. (1987). Biochemical characteristics of isolated rat liver stellate cells. Hepatology.

[27] Hendriks H.F., Brekelmans P.J., Buytenhek R., Brouwer A., de Leeuw A.M., Knook D.L. (1987). Liver parenchymal cells differ from the fat-storing cells in their lipid composition. Lipids.

[28] Moriwaki H., Blaner W.S., Piantedosi R., Goodman D.S. (1988). Effects of dietary retinoid and triglyceride on the lipid composition of rat liver stellate cells and stellate cell lipid droplets. J. Lipid Res..

[29] Mayer N., Schweiger M., Romauch M., Grabner G.F., Eichmann T.O., Fuchs E., Ivkovic J., Heier C., Mrak I., Lass A., Hofler G., Fledelius C., Zechner R., Zimmermann R., Breinbauer R. (2013). Development of small-molecule inhibitors targeting adipose triglyceride lipase. Nat. Chem. Biol..

[30] Schweiger M., Schreiber R., Haemmerle G., Lass A., Fledelius C., Jacobsen P., Tornqvist H., Zechner R., Zimmermann R. (2006). Adipose triglyceride lipase and hormone-sensitive lipase are the major enzymes in adipose tissue triacylglycerol catabolism. J. Biol. Chem..

[31] Bartz R., Zehmer J.K., Zhu M., Chen Y., Serrero G., Zhao Y., Liu P. (2007). Dynamic activity of lipid droplets: protein phosphorylation and GTP-mediated protein translocation. J. Proteome Res..

[32] Schweiger M., Lass A., Zimmermann R., Eichmann T.O., Zechner R. (2009). Neutral lipid storage disease: genetic disorders caused by mutations in adipose triglyceride lipase/PNPLA2 or CGI-58/ABHD5. Am. J. Physiol. Endocrinol. Metab..

[33] Ong K.T., Mashek M.T., Bu S.Y., Greenberg A.S., Mashek D.G. (2011). Adipose triglyceride lipase is a major hepatic lipase that regulates triacylglycerol turnover and fatty acid signaling and partitioning. Hepatology.

[34] Wu J.W., Wang S.P., Alvarez F., Casavant S., Gauthier N., Abed L., Soni K.G., Yang G., Mitchell G.A. (2011). Deficiency of liver adipose triglyceride lipase in mice causes progressive hepatic steatosis. Hepatology.

[35] Chandak P.G., Radovic B., Aflaki E., Kolb D., Buchebner M., Frohlich E., Magnes C., Sinner F., Haemmerle G., Zechner R., Tabas I., Levak-Frank S., Kratky D. (2010). Efficient phagocytosis requires triacylglycerol hydrolysis by adipose triglyceride lipase. J. Biol. Chem..

[36] Zechner R., Zimmermann R., Eichmann T.O., Kohlwein S.D., Haemmerle G., Lass A., Madeo F. (2012). FAT SIGNALS–lipases and lipolysis in lipid metabolism and signaling. Cell Metab..

[37] Gao J., Simon M. (2005). Identification of a novel keratinocyte retinyl ester hydrolase as a transacylase and lipase. J. Investig. Dermatol..

[38] Gao J.G., Simon M. (2007). A comparative study of human GS2, its paralogues, and its rat orthologue. Biochem. Biophys. Res. Commun..

[39] Kluwe J., Wongsiriroj N., Troeger J.S., Gwak G.Y., Dapito D.H., Pradere J.P., Jiang H., Siddiqi M., Piantedosi R., O'Byrne S.M., Blaner W.S., Schwabe R.F. (2011). Absence of hepatic stellate cell retinoid lipid droplets does not enhance hepatic fibrosis but decreases hepatic carcinogenesis. Gut.

[40] Tsutsumi C., Okuno M., Tannous L., Piantedosi R., Allan M., Goodman D.S., Blaner W.S. (1992). Retinoids and retinoid-binding protein expression in rat adipocytes. J. Biol. Chem..

[41] Osuga J., Ishibashi S., Oka T., Yagyu H., Tozawa R., Fujimoto A., Shionoiri F., Yahagi N., Kraemer F.B., Tsutsumi O., Yamada N. (2000). Targeted disruption of hormone-sensitive lipase results in male sterility and adipocyte hypertrophy, but not in obesity. Proc. Natl. Acad. Sci. U. S. A..

[42] Zimmermann R., Haemmerle G., Wagner E.M., Strauss J.G., Kratky D., Zechner R. (2003). Decreased fatty acid esterification compensates for the reduced lipolytic activity in hormone-sensitive lipase-deficient white adipose tissue. J. Lipid Res..

[43] Strom K., Hansson O., Lucas S., Nevsten P., Fernandez C., Klint C., Moverare-Skrtic S., Sundler F., Ohlsson C., Holm C. (2008). Attainment of brown adipocyte features in white adipocytes of hormone-sensitive lipase null mice. PLoS One.

[44] Shen W.J., Yu Z., Patel S., Jue D., Liu L.F., Kraemer F.B. (2011). Hormone-sensitive lipase modulates adipose metabolism through PPARgamma. Biochim. Biophys. Acta.

[45] Haemmerle G., Moustafa T., Woelkart G., Buttner S., Schmidt A., van de Weijer T., Hesselink M., Jaeger D., Kienesberger P.C., Zierler K., Schreiber R., Eichmann T., Kolb D., Kotzbeck P., Schweiger M., Kumari M., Eder S., Schoiswohl G., Wongsiriroj N., Pollak N.M., Radner F.P., Preiss-Landl K., Kolbe T., Rulicke T., Pieske B., Trauner M., Lass A., Zimmermann R., Hoefler G., Cinti S., Kershaw E.E., Schrauwen P., Madeo F., Mayer B., Zechner R. (2011). ATGL-mediated fat catabolism regulates cardiac mitochondrial function via PPAR-alpha and PGC-1. Nat. Med..

[46] Ahmadian M., Abbott M.J., Tang T., Hudak C.S., Kim Y., Bruss M., Hellerstein M.K., Lee H.Y., Samuel V.T., Shulman G.I., Wang Y., Duncan R.E., Kang C., Sul H.S. (2011). Desnutrin/ATGL Is Regulated by AMPK and Is Required for a Brown Adipose Phenotype. Cell Metab..

[47] Lee U.E., Friedman S.L. (2011). Mechanisms of hepatic fibrogenesis. Best Pract. Res. Clin. Gastroenterol..

[48] Mormone E., George J., Nieto N. (2011). Molecular pathogenesis of hepatic fibrosis and current therapeutic approaches. Chem. Biol. Interact..

[49] Shirakami Y., Lee S.A., Clugston R.D., Blaner W.S. (2012). Hepatic metabolism of retinoids and disease associations. Biochim. Biophys. Acta.

[50] Davis B.H., Coll D., Beno D.W. (1993). Retinoic acid suppresses the response to platelet-derived growth factor in human hepatic Ito-cell-like myofibroblasts: a post-receptor mechanism independent of raf/fos/jun/egr activation. Biochem. J..

[51] Hellemans K., Verbuyst P., Quartier E., Schuit F., Rombouts K., Chandraratna R.A., Schuppan D., Geerts A. (2004). Differential modulation of rat hepatic stellate phenotype by natural and synthetic retinoids. Hepatology.

[52] Mizobuchi Y., Shimizu I., Yasuda M., Hori H., Shono M., Ito S. (1998). Retinyl palmitate reduces hepatic fibrosis in rats induced by dimethylnitrosamine or pig serum. J. Hepatol..

[53] Seifert W.F., Bosma A., Brouwer A., Hendriks H.F., Roholl P.J., van Leeuwen R.E., van Thiel-de Ruiter G.C., Seifert-Bock I., Knook D.L. (1994). Vitamin A deficiency potentiates carbon tetrachloride-induced liver fibrosis in rats. Hepatology.

[54] Geubel A.P., De Galocsy C., Alves N., Rahier J., Dive C. (1991). Liver damage caused by therapeutic vitamin A administration: estimate of dose-related toxicity in 41 cases. Gastroenterology.

[55] Dan Z., Popov Y., Patsenker E., Preimel D., Liu C., Wang X.D., Seitz H.K., Schuppan D., Stickel F. (2005). Hepatotoxicity of alcohol-induced polar retinol metabolites involves apoptosis via loss of mitochondrial membrane potential. FASEB J..

[56] Jha P., Claudel T., Baghdasaryan A., Mueller M., Halilbasic E., Das S.K., Lass A., Zimmermann R., Zechner R., Hoefler G., Trauner M. (2014). Role of adipose triglyceride lipase (PNPLA2) in protection from hepatic inflammation in mouse models of steatohepatitis and endotoxemia. Hepatology.

